# The Quest for Genes Involved in Adaptation to Climate Change in Ruminant Livestock

**DOI:** 10.3390/ani11102833

**Published:** 2021-09-28

**Authors:** Matilde Maria Passamonti, Elisa Somenzi, Mario Barbato, Giovanni Chillemi, Licia Colli, Stéphane Joost, Marco Milanesi, Riccardo Negrini, Monia Santini, Elia Vajana, John Lewis Williams, Paolo Ajmone-Marsan

**Affiliations:** 1 Department of Animal Science, Food and Nutrition—DIANA, Università Cattolica del Sacro Cuore, Via Emilia Parmense, 84, 29122 Piacenza, Italy; matildemaria.passamonti1@unicatt.it (M.M.P.); elisa.somenzi@unicatt.it (E.S.); mario.barbato@unicatt.it (M.B.); licia.colli@unicatt.it (L.C.); riccardo.negrini@unicatt.it (R.N.); johnlewis.williams@unicatt.it (J.L.W.); 2 Department for Innovation in Biological, Agro-Food and Forest Systems–DIBAF, Università Della Tuscia, Via S. Camillo de Lellis snc, 01100 Viterbo, Italy; gchillemi@unitus.it (G.C.); marco.milanesi@unitus.it (M.M.); 3 Research Center on Biodiversity and Ancient DNA—BioDNA, Università Cattolica del Sacro Cuore, Via Emilia Parmense, 84, 29122 Piacenza, Italy; 4 Laboratory of Geographic Information Systems (LASIG), School of Architecture, Civil and Environmental Engineering (ENAC), Ecole Polytechnique Fédérale de Lausanne (EPFL), 1015 Lausanne, Switzerland; stephane.joost@epfl.ch (S.J.); elia.vajana@epfl.ch (E.V.); 5 Impacts on Agriculture, Forests and Ecosystem Services (IAFES) Division, Fondazione Centro Euro-Mediterraneo Sui Cambiamenti Climatici (CMCC), Viale Trieste 127, 01100 Viterbo, Italy; monia.santini@cmcc.it; 6 Nutrigenomics and Proteomics Research Center—PRONUTRIGEN, Università Cattolica del Sacro Cuore, Via Emilia Parmense, 84, 29122 Piacenza, Italy

**Keywords:** climate change, livestock, adaptation

## Abstract

**Simple Summary:**

After domestication in specific regions, livestock followed human migrations and colonized the whole world. During this population expansion, human and natural selection, together with demographic events, molded the livestock genome leading to local breeds and populations able to produce milk, meat, wool and tractive power in many different agro-climatic conditions. The climate is changing, with temperatures and the frequency of extreme climatic events increasing, which affects livestock welfare and production efficiency, particularly of the highly productive breeds. Genomics is now able to explore the DNA of local breeds adapted to extreme environments in search of genes carrying signatures of selection for adaptation. This review summarizes methods used to accomplish this task, giving examples of results achieved and perspectives for future breeding.

**Abstract:**

Livestock radiated out from domestication centres to most regions of the world, gradually adapting to diverse environments, from very hot to sub-zero temperatures and from wet and humid conditions to deserts. The climate is changing; generally global temperature is increasing, although there are also more extreme cold periods, storms, and higher solar radiation. These changes impact livestock welfare and productivity. This review describes advances in the methodology for studying livestock genomes and the impact of the environment on animal production, giving examples of discoveries made. Sequencing livestock genomes has facilitated genome-wide association studies to localize genes controlling many traits, and population genetics has identified genomic regions under selection or introgressed from one breed into another to improve production or facilitate adaptation. Landscape genomics, which combines global positioning and genomics, has identified genomic features that enable animals to adapt to local environments. Combining the advances in genomics and methods for predicting changes in climate is generating an explosion of data which calls for innovations in the way big data sets are treated. Artificial intelligence and machine learning are now being used to study the interactions between the genome and the environment to identify historic effects on the genome and to model future scenarios.

## 1. Introduction 

**Figure 1:**
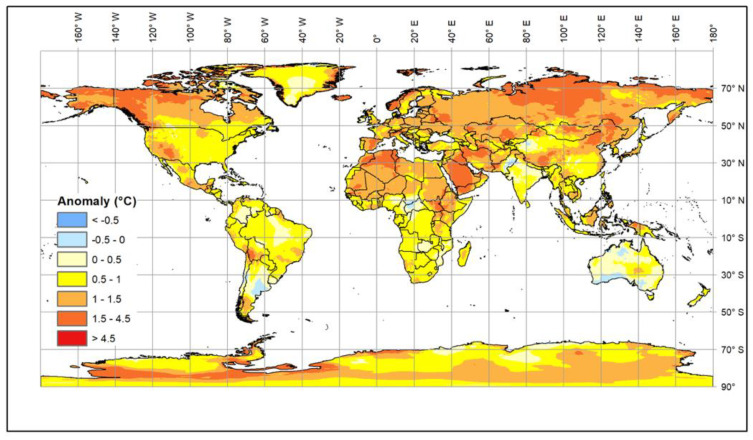
Map of annual temperature changes in the period 1992–2020 compared to 1950–1978, created using ERA5 climate Reanalysis tools. The areas showing warming are in yellow-red and those showing cooling are in blue.

Climate change is generally causing an increase in global temperatures (see Box 1). The most recent estimates [1] suggest that a 1.5 °C warming compared to the 1850–1900 baseline will be reached in the second half of the current decade, but, in addition, there are longer cold periods and increased levels of solar radiation [2,3,4,5,6] (Figure 1).

These changes affect both extensive and intensive farming systems [7,8]. The impact of environmental changes on animals affects their health, growth, and fertility as well as the diseases to which they are exposed. In addition, availability and types of feed may change because of the impact of the climate on the production and quality of grains, pasture and forage crops [9,10], which will affect nutrition as well as animal health and metabolism [11]. Livestock can adapt to gradual changes in environmental temperature. However, rapid changes or extended periods of extreme conditions reduce their welfare and productivity and are potentially life threatening. Therefore, the current rapid rise in global temperature is increasingly exposing livestock to stress in many countries. Some local breeds that have been kept in areas with adverse conditions, such as high temperature and humidity or drought, have become adapted over many generations; these breeds are an invaluable resource for research and breeding. It is urgent to understand the biological mechanisms underlying their adaptability, and, in particular, to identify genomic regions and genes that control such mechanisms in order to facilitate the rapid selection of livestock resilient to climate change. This review focuses on ruminants and on the current state of knowledge on genetics controlling adaptation.

## 2. Impacts of Climate Change on Livestock

With increasing global temperatures, more productive livestock are at greater risk (see Box 2), because they have higher feed intake and feed consumption, which is directly related to animal heat production [12]. Animals eat less to counteract high temperatures, and nutrients are prioritized to support maintenance rather than production and reproduction. In the central U.S., for example, severe losses of beef cattle kept in feedlots have been reported because of heat waves in summer and extreme snowstorms and wind in winter [13]. Climate related economic losses as a result of animal death and reduced performance have been seen [14]. Cattle, sheep, pigs and chickens reduce their feed intake by 3–5% for each unit increase in temperature above 30 °C [15]. Reproduction is particularly affected. Hahn [16] reported that conception rates in dairy cows are reduced by 4.6% per unit change above 70 in the temperature humidity index (THI) [17]. For beef cattle kept in range or pasture management systems, a decrease in pregnancy rates of 3.2% and 3.5% was observed for each unit increase in average THI above 70 and an increase in average temperature above 23.4 °C, respectively. Among environmental variables, temperature has the greatest effect on cow pregnancy rates [18]. 

Climate change further includes altered rainfall patterns that, combined with geographical factors such as soil type, affect crop production [19,20,21]. Drought reduces biomass [22], increases lignin accumulation in plant tissues, and reduces proteins, resulting in less digestible forages [23] and insufficient energy to meet livestock requirements [24,25]. Increased occurrence of prolonged drought is therefore of great concern to pasture-based livestock systems [23], especially those in environments which cannot support arable production [26].

Climate change influences the distribution of animal pathogen vectors and parasite range [27] which, together with the decreased immune response of animals under stress (triggered by cortisol), exposes livestock to higher risks of disease. Early springs, warmer winters and changes in rainfall distribution affect the seasons in which pathogens, parasites and vectors are present, potentially increasing proliferation and survival of these organisms. Bluetongue recently spread northward from Africa to Europe [28] as a consequence of climate-driven ecosystem changes and the associated expansion of the geographic range of the insect *Culicoides imicola*, the vector of the virus [29]. Other vectors such as the tick *Rhipicephalus appendiculatus,* which is the host for the protozoan pathogen *Theileria parva*, are predicted to shift their geographic range due to climate change, moving southward from central sub-Saharan Africa towards southern Africa [30]. Higher temperatures in Europe have increased parasite burdens such as helminths, with a shift from species traditionally found in temperate zones such as *Ostertagia ostertagi* to tropically adapted species, particularly *Haemonchus contortus* [31,32]. In addition to temperature, increased rainfall and humidity have affected the distribution of parasites. Leptospirosis in humans has been linked to transmission from livestock, with many outbreaks reported following extreme weather events around the world [33].

Box 1 **Climate Data and Tools.**

High resolution meteorological data are used to evaluate climate trends and variability and to predict the frequency of extreme events. Where meteorological data are not available, advanced climate modelling produces “Climate Reanalysis” datasets for a comprehensive description of the climate in three-dimensional grids. “Climate Reanalysis” has become an essential tool for modelling meteorological data to provide services to sectors dependent on climate assessments, forecasts and projections, including ecosystem management, agriculture, and livestock farming [34,35]. Climate modelling is also able to produce short- to long-term climate predictions (months to a few decades ahead), and projections extending over many decades at the global level.

Bioclimatic indicators allow the ever–increasing climate datasets to be combined and condensed and are valuable for both expert and non-expert users. Bioclimatic indicators from several global datasets are available from WorldClim [36], CHELSA [37], CliMond [38], ecoClimate [39], ENVIREM [40], MERRAclim [41], CMCC-BioclimInd [42] and the latest, KGClim [43]. The FAO (Food and Agriculture Organization of the United Nations), provides Global Agro-Ecological Zoning (GAEZ) indicators of the likely variation in agricultural resources over time. Agrometeorological indicators from 1979 to the present and agro-climatic indicators from 1951 to 2099 derived from Climate Reanalysis and projections are available from Climate Change Service (C3S) of the Copernicus programme [44]. Frequency, duration, timing and severity of extreme weather events can be calculated using indicators and indices for climate extremes such as those defined by the Expert Team on Climate Change Detection Monitoring and Indices (ETCCDI) [45,46].

## 3. Becoming Adapted

Archaeological evidence and molecular analysis of present-day DNA variation suggest that livestock were domesticated in specific regions of different continents. The Fertile Crescent region in Southwest Asia is one of these. Here the wild progenitors of cattle, sheep, goats and pigs progressively adapted to a closer relationship with humans and finally became dependent on human care. Archaeozoological and mitochondrial DNA diversity data have confirmed that domestication of these species occurred in a climatically homogeneous area around the Fertile Crescent, comprising South-eastern Anatolia and the Iranian Zagros Mountains [47,48,49]. After domestication, livestock followed human migrations and, with agricultural expansion, colonized the whole world [50,51]. Technological advances have facilitated the study of ancient DNA (aDNA) from well–preserved archaeological remains, which is shedding light on the spatiotemporal dynamics of domestication and on the physiological and neurobiological changes that livestock species underwent during the transition from the wild to a domestic existence, as well as on the subsequent adaptation to different environments and selection for functional traits [52]. For example, these studies, have shown that cattle and goat domestication took place over relatively large geographical areas and extended time frames [53,54,55], with frequent events of admixture and introgression, sometimes from several wild relative species [53,56]. Over millennia livestock species have adapted to thrive in a range of environments, with different temperature, humidity, water and fodder availability and quality, pathogen and parasite challenges, and also to satisfy human needs for food, wool, fibre and tractive power.

At the genetic level, signals of adaptive changes driven by domestication have been found in genes related to nervous system development [57,58] including kit ligand (*KITLG*), the treacle ribosome biogenesis factor *1* (*TCOF1*), and fibroblast growth factor receptor 1 (*FGFR1*) [57]. Other signatures of selection, or of adaptive introgression from wild relatives, have been found in genes implicated in adaptation to feed and farming regimes. A variant in the cytochrome P450 2C19 gene (*CYP2C19*) has been under positive selection in goats. *CYP2C19* is a member of the *CYP2C* subfamily of the cytochrome P450 superfamily of genes [59] which confers protection against a mycotoxin produced by Fusarium spp. fungi in cereals [54]. Therefore, the increased frequency of the variant is most likely a response to an increasingly cereal-based diet contained in waste by-products. Alleles that may have been introgressed into domesticated goats from *Capra caucasica* [60], a West Caucasian tur–like species, have been found in a genomic region harbouring genes that affect immune function and parasite resistance, including *SERPINB3, SERPINB4, CD1B, COL4A4, BPI, MAN2A1*, and *CD2AP*. In particular, the mucin 6 oligomeric mucus/gel–forming gene (*MUC6*), which encodes a gastro-intestinally secreted mucin, is nearly fixed in goats for the Tur–derived haplotype, which confers enhanced immune resistance to gastrointestinal pathogens [56]. The fixation of this introgressed variant may be the consequence of the adaptive advantage it provided in farm environments, where there is increased exposure to parasites and disease [56].

Recently, the characterization of the paleo-epigenome and paleo-microbiomes of domestic species have facilitated the exploration of their role in the adaptation of mammalian livestock to their environment [61]. Data on the epigenomic profiles or microbiota composition in ancient livestock may provide information on diet, lifestyle, health status and exposure to stressors, and thus help us to explore the mechanisms of adaptation and interaction with the environment on a micro-evolutionary scale.

Animals adapt to the environments in which they live and to external stress by acclimation to a particular stressor or to a range of stressors [62,63]. Adaptation can be crucial for survival, but often negatively affects the productivity and profitability of livestock systems. The ability to adapt depends in part on the flexibility of behavioral traits [64] and in part on morphological and physiological changes that better adapt animals for survival. For example, about 25% of sheep in the world are fat tail or fat rump breeds that are adapted to harsh semi-arid desert conditions where food availability is sporadic. The fat tail or rump acts as a store, to enable the animals to survive long periods when food is in short supply [65].

Cattle adapted to prolonged heat stress have increased hemoglobin and red cell numbers [66], which may also protect them against blood borne parasites such as theileriosis. *Bos taurus taurus* cattle that have been raised over many generations in cool and temperate climates have long hair, subcutaneous fat, and often a dark coat colour. In contrast, *Bos taurus indicus* cattle that were originally from hotter tropical climates have short hair, little subcutaneous fat, low metabolism, and a body conformation to aid heat dispersion, with high surface to volume ratio, large ears and loose skin, especially around the dewlap [67,68]. To increase performance while maintaining environmental resilience, crosses between taurine and indicine cattle have been developed [69]. The crossbred animals show better adaptation to high temperature and humidity, and to parasites, e.g., resistance to *Boophilus microplus* ticks increases in proportion to *Bos taurus indicus* ancestry in the cross [70].

Box 2 **Heat Stress Indicators.**

The level of heat stress experienced by an animal is the result of a combination of air temperature, relative humidity [71] and other climate factors including wind speed and solar radiation [72]. Depending on the management system, these parameters may make different contributions to the risk of thermal stress [73]. Environmental parameters can be measured and used to construct indices and set thresholds to define risk situations.

Most of the indices defining thermal stress risk have been developed for cattle, especially for dairy cows that are particularly susceptible to high temperatures. The Temperature Humidity Index (THI) [17] takes into account the effect of air temperature and humidity. THI was originally developed as a general indicator of heat stress for humans, but today is also applied to livestock. Over the years, the model and threshold values used to define heat stress conditions have been modified [73], and corrections are now applied if cooling systems are used in the housing [74]. THI does not take into account the cumulative effect of high temperature [73] or the impact of wind speed and solar radiation, which are important when estimating the level of heat stress experienced by an animal. The Equivalent Temperature Index (ETI) includes air speed in the formula [75], although solar radiation is not considered [73]. The THI adjusted (THIadj) index considers both the wind speed and the solar radiation, as well as breed and coat colour [76]. The Respiration Rate index (RR) is an extension of THIadj that also takes into account whether animals are in a shaded area or under the sun [77].

Other prediction models that have been developed to overcome the limitations of THI include the heat load index (HLI), which incorporates “black globe” temperature measurements substituting air temperature, animal factors (genotype, coat colour and health status) and management strategies (shade availability, days on feed, manure management and temperature of drinking water). These factors are used to modify the threshold to define the heat stress, and combined with factors to account for location-specific variables in different geographic areas [78]. HLI is considered a better predictor than THI as it includes the interaction between climatic variables and animal thermal exchange mechanisms [78]. The Accumulate Heat Load Unit (AHLU) index, based on HLI, is a measure of the animal’s heat load balance [79]. The AHLU may increase or decrease over time depending on HLI values. A zero AHLU value indicates that the animal is in thermal balance [79]. The HLI has also been extended to create a Comprehensive Climate Index (CCI) that can also be used under cold conditions [80].

A comprehensive review of models for predicting heat stress response in livestock is given in Rashamol et al. [78].

Senepol cattle were developed on the island of St Croix to create a breed that was polled, easily managed and tolerant of the tropical environment by crossing red polled taurine cattle with African Zebu cattle [81]. Some of these cattle have very short hair and reduced follicle density, giving the phenotype referred to as “SLICK”. SLICK is controlled by a single genetic locus and carriers of the Slick variant have lower core temperature than non-SLICK contemporaries [82]. Interestingly, the effect of SLICK is most likely through increased sweat production rather than the decrease in hair length and density [83]. The SLICK variant in Senepol cattle was initially mapped to chromosome 20 [84], and later the causative variation was identified in the prolactin receptor gene (*PRLR*). A single base deletion in exon 10 causes a frameshift that introduces a stop codon and results in the truncation of the protein [85]. Other criollo cattle breeds, such as Carora and Limonero, that were brought to the Americas from Spain 500 years ago [86] display a similar SLICK phenotype. However, these breeds do not carry the same prolactin variant that was identified in the Senepol cattle, although a genome-wide association analysis located the causative variant in or near to *PLRL*. DNA sequencing of SLICK Limonero cattle revealed three variants within the prolactin receptor gene that create premature stop codons in exon 11, one of which is also found in SLICK Carora cattle [87]. Recently, three novel variants were discovered in the *PLRL* gene in six Caribbean Basin cattle breeds. All create premature stop codons and increase heat tolerance. The occurrence of mutations in the prolactin receptor in several cattle breeds that are adapted to tropical climates and that have distinct evolutionary histories is unlikely to be by chance. Indeed, prolactin levels have been shown to be involved in thermoregulation in humans [88], showing that certain physiological processes and specific genes can be targeted by environmental pressure. The SLICK variant has now been introgressed into other breeds, including the highly productive Holstein dairy breed, creating more heat tolerant animals [89].

Nevertheless, adaptation generally requires changes in the combination of alleles of many genes; for example, the genomic analysis of admixture between *Bos taurus taurus* and Zebu (*Bos taurus indicus* cattle) in Africa showed that more than 150 loci were under selection for local adaptation [90]. The ability of livestock to successfully adapt to extreme climatic conditions and to tolerate a wide range of parasites has resulted in local populations with specific characteristics. These populations are valuable resources that, if well characterized, could be exploited to create breeds suited to new conditions arising from climate change.

Box 3 **The Genome and Genomics.**

The publication of the human genome sequence in 2001 [91] was a landmark that opened new opportunities in molecular genetics. The same approach that was used to sequence the human genome was used to produce draft sequences for the major livestock species; the first was the chicken in 2004 [92], followed by the cow in 2009 [93], then the pig [94], sheep [95] and goat [96] in 2012. These genomes became references against which DNA and RNA sequences from these species were aligned and compared. With the rapidly advancing sequencing technologies, which progressed from automated Sanger sequencing to next-generation high throughput short read sequencing [97], large numbers of individuals were sequenced at low resolution. Alignment of these sequences with the reference genomes revealed huge numbers of variations among individuals, in particular, Single Nucleotide Polymorphisms (SNP). This SNP data led to the development of genome-wide genotyping panels. A range of low (few thousand) to high (many hundred thousand) density SNP panels is commercially available, including some targeted to specific traits, and others that include SNP for several species to reduce costs of genotyping. Knowledge of the genome sequence from large numbers of individuals in a population enables low density SNP genotype data to be used to estimate higher density genotypes by “imputation” [98].

The analysis of phenotype and genotype in genome-wide association studies enables genetic loci with a major effect on the phenotype to be identified (e.g., [99,100,101]). In some cases the genes and causative polymorphisms controlling variations in target traits have been identified (e.g., [102]). Perhaps the most important advance coming from the availability of genome-wide SNP panels is that the idea of genome-based selection envisioned by Meuwissen and colleagues more than a decade ago has now been realized [103]. Other applications of the SNP panels include the analysis of population structure, history and diversity (e.g., [104,105,106] to guide conservation strategies [107] and the identification of regions of the genome that are under selection (e.g., [108]).

Next generation sequencing (NGS) has also facilitated the study of gene expression by enabling the analysis of the whole transcriptome [109]. Depending on how samples are processed and analysed, this approach can examine the expression of genes (e.g., [110,111]), variations in splice sites [112], and non-coding RNAs [113,114] as well as short, micro-RNAs [115] that have a regulatory role.

Further advances in sequencing technology are opening new opportunities. Long read, single molecule sequencing has enabled haplotype resolved genome sequences to be produced by separating the sequence reads originating from the maternally and paternally inherited chromosome [116,117]. Long read technologies such as Pacific Biosciences and Oxford Nanopore can produce full length sequences of transcripts to reveal isoforms present in different tissues or diverse physiological states. These technologies are also able to distinguish modified bases in the DNA, specifically methylation, in order to examine epigenetic patterns directly and explore the regulation of gene expression [118]. The Functional Annotation of Animal Genomes Consortium [119] is assembling data on genome structure, expression, and regulation using a range of new technologies. For an extensive review of the state of livestock genomics see Georges et al. [120].

## 4. Seeking Adaptive Genes

Several molecular genetic approaches have been used to identify adaptation-related genes. Genome wide association studies (GWAS) use phenotypes related to adaptation recorded directly on the animals. Landscape Genomics approaches use environmental variables as proxies for phenotypes. Other methods analyse the patterns of genomic diversity within and between populations and the level of admixture in specific genomic regions to identify selection signatures of adaptation. These approaches use genomic tools that may focus on individual loci through to whole genomic sequence analyses (see Box 3) and dedicated software (Table 1).

### 4.1. Genome-Wide Association Studies

Genome-wide association studies (GWAS) identify the association between variations in the genome, the genotype, with variations in phenotype displayed by individual animals belonging to a same breed or population. GWAS therefore requires both genotype and phenotype data on each individual [121,122]. Fulfilling such conditions is difficult for complex phenotypes, and not always feasible when the target population is small or isolated [123], which is often the case in adaptation studies. Moreover, costs for genotyping and trait recording represents a further hurdle in reaching an adequate sample size. For these reasons, GWAS carried out in livestock to understand the genetic control of complex traits, are invariably low powered and results between studies on the same traits are often inconsistent. In addition, the genetic associations identified are likely to differ depending on the way that a trait is measured, the genetic background and the environment. Livestock GWAS have primarily been used to identify genetic variants associated with specific production traits or disease responses [124]. GWAS that identify the genes controlling climate adaptation traits (e.g., efficient thermoregulation, feed utilization, and immunity) would accelerate selection for animals more resilient to climatic challenges [125].

Several statistical tests have been applied to identify marker–trait associations in GWAS, from single marker regression, to mixed model and Bayesian approaches that use different marker effect distributions as prior information, to haplotype based GWAS [126]. In all cases, corrections have to be applied for multiple testing and for population structure in order to avoid a high number of false positives. As most traits involved in adaptation are highly complex and have a low to moderate heritability, a large cohort of animals has to be investigated to reach a sufficient statistical power in GWAS. [127,128].

A GWAS of cattle indigenous to Benin [99] identified several potential candidate genes associated with stress and immune response (*PTAFR, PBMR1, ADAM, TS12*), feed efficiency (*MEGF11, SLC16A4, CCDC117*), and conformation and growth (*VEPH1, CNTNAP5, GYPC*). The study of cold stress in Siberian cattle breeds identified two candidate genes (*MSANTD4* and *GRIA4*) on chromosome 15, putatively involved in cold shock response and body thermoregulation [100]. GWAS in taurine, indicine and cross-bred cattle identified *PLAG1* (BTA14), *PLRL* (BTA20) and *MSRB3* (BTA5) as candidate genes for several traits important for adaptation to extensive tropical environments [101]. A GWAS of the Frizarta dairy sheep breed, which is adapted to a high relative humidity environment, identified 39 candidate genes associated with body size traits including *TP53, BMPR1A, PIK3R5, RPL26,* and *PRKDC* [129]. An association analysis of genotype-by-environment (GxE) interactions with growth traits in Simmental cattle showed that birth weight was affected by temperature, while altitude affected weaning and yearling weight. Genes implicated in these traits included neurotransmitters (*GABRA4* and *GABRB1*), hypoxia-induced processes (*PLA2G4B, PLA2G4E, GRIN2D,* and *GRIK2)* and keratinization (*KRT15, KRT31, KRT32, KRT33A, KRT34,* and *KRT3)*, all processes that play a role in physiological responses associated with adaptation to the environment [130].

Enhancing efficiency would reduce the impact of changes in feed availability on livestock systems and potentially reduce methane production, which contributes to climate change. Residual feed intake (RFI), that is, the difference between actual feed intake and the theoretical energy requirements of an animal [131], has been used to select for increased feed efficiency (FE) [132,133]. A GWAS of RFI in Nellore cattle identified QTL on chromosomes 8 and 21 affecting the trait. Putative candidate genes on BTA 8 are *CCDC171* and *CLCN3* [134], while candidates on BTA11 are *DEPP1,* expression of which is induced by fasting, *TUBB3* and *PTSG1* [135].

A GWAS for temperament scores carried out on crossbred steers in a feedlot identified five SNP on BTA 1, 24, and 29 and 13 SNP on BTA11 [136]. Functional candidate genes close to these loci had roles in neural function included synaptotagmin 4 (BTA 24), FAT atypical cadherin 3 (BTA 29), tubulin tyrosine ligase-like 1 (BTA 5), spermatogenesis associated 17 (BTA 16), stanniocalcin 2 (BTA 20), and GABAA receptor γ 3 (BTA 21). A GWAS of 3,274 Charolais beef cows detected four significant and 12 suggestive chromosomal regions associated with several functional and behavioral traits including aggressiveness [137]. A recent GWAS analysis of 1,370 Brahman cattle clustered in two groups of temperament identified nine SNP located in intergenic regions near candidate genes *ACER3*, *VRK2*, *FANCL* [138].

### 4.2. Selection Signatures

Natural or artificial selective pressure causes an increase or decrease in the frequency of genetic variants in a population. Selection can be positive, balancing, or negative [139]. Positive selection increases the frequency of fitness-enhancing variants in a population whereas negative selection removes unfavourable mutations to restore DNA functional integrity [140]. Balancing selection retains more than one allele of a gene where heterozygotes have higher fitness [141]. The genes in the genomic region in linkage disequilibrium with the genes under selection will also increase or decrease in frequency through the hitch-hiker effect [142], changing the expected patterns of molecular variation and giving a “selection signature”.

Tajima’s D statistic (See Box 4) has been used to analyse wild and domestic sheep data to identify a genomic region involved in the resistance to pneumonia [143]. A scan of Russian cattle genomes using Tajima’s D statistic detected signatures of selection most likely resulting from adaptation to cold environments [144]. Fay and Wu’s H statistic has been used with cattle data to detect signals of recent positive selection involving genes associated with innate immune response [145].

Signatures of recent selection associated with aggressiveness have been identified on chromosome X by comparing the Lidia cattle breed, which has been selected for aggressive responses, with two Spanish breeds showing docile behaviour. The most significant selection signature included the monoamine oxidase A gene (*MAOA*) [146]. A further refinement of the analysis identified a variable number of tandem repeats in the gene, with the Lidia breed having fewer repeats compared with the docile breeds [147]. Favourable genetic and phenotypic relationships between docility and meat quality, feedlot performance, ease of transport and reproductive traits have been reported [148]. Temperamental animals generally are not as well adapted to stress and have slow growth rates, poor carcass conformation and poor immune function [149,150]. Differences in docility have also been found between *Bos taurus taurus* and *Bos taurus indicus* cattle (e.g., [151] and between beef and dairy breeds [152].

Signatures of selection related to feed adaptation have been found in sheep using an F_ST_ approach [153]. Of the seventeen genes under climatic selection, nine were related to energy metabolism. The strongest selection signal was around *TBC1D12,* on OAR22, which plays a role in GTPase regulation. The F_ST_ approach was also applied to Siberian cattle populations in order to understand the genetic basis of adaptation to cold environments [154]. Results identified several genes that have been implicated in thermal adaptation in cattle, such as *GRIA4*, *COX17*, *MAATS1*, *UPK1B*, *IFNGR1*, *DDX23*, *PPT1*, *THBS1*, *CCL5*, *ATF1*, *PLA1A*, *PRKAG1,* and *NR1I2.*

With regard to hot environments, Li and colleagues [155] investigated selection signatures of bovine heat tolerance in Dehong cattle, a Chinese indigenous zebu breed, using an F_ST_ approach. Results indicated that genes involved in heat shock (*HSF1*), oxidative stress response (*PLCB1*, *PLCB4*), coat color (*RAB31*), feed intake (*ATP8A1*, *SHC3*) and reproduction (*TP63, MAP3K13, PTPN4, PPP3CC, ADAMTSL1, SS18L1, OSBPL2, TOX, RREB1,* and *GRK2*) may play a role in heat adaptation.

Pairwise comparison of genetic differentiation of sheep breeds adapted to different environments identified selection signatures in the genes *MITF, FGF5, MTOR, TRHDE* and *TUBB3* that have been associated with high-altitude adaptation [156]. An F_ST_ statistic approach applied to cattle breeds reared in different environments identified several genes under positive selection for thermal tolerance [157]. HapFLK detected the Nebulin Related Anchoring Protein gene (*NRAP)* to be under selection for adaptation to cold environments [158], *ACSS2, ALDOC, EPAS1, EGLN1* and *NUCB2* to be under selection for high-altitude adaptation in cattle [159], and *DNAJC28, GNRH1* and *MREG* to be associated with heat stress adaptation in sheep [160]. 

iHS methods have been used to detect signatures of adaptation to environments in French Charolais cattle, sheep and goats [161,162,163,164,165]. Cross-population EHH-based tests have been used to detect hot climate adaptation in cattle [166,167,168] and sheep [169,170,171], and hypoxia adaptation in new world camelids [172]. Detecting runs of homozygosity (ROHs) to find regions containing genes associated with adaptation has been demonstrated in several domestic species [157,162,163,173,174].

Box 4 **Approaches for Selection Signature Detection.**

Selection on a locus, whether artificial for production or natural for adaptation, is associated with the reduction of genetic diversity in the region, creating a “selection signature”. Tajima’s test [175] is able to detect positive selection sweeps that occurred recently, as it identifies regions with high numbers of rare, low-frequency variants that are the result of recent mutation [176]. Fay and Wu statistics [177], in contrast, assess the relationship between ancestral and derived alleles, which enables both positive and negative recent selection occurring in medium- to high-frequency alleles to be detected. However, knowledge of ancestral alleles is necessary to apply the method [178].

Various approaches have been used to assess positive and negative selection in populations. Wright’s fixation index (F_ST_) measures differences in allele frequencies between populations based on individual loci. F_ST_ has been used in many studies of livestock to explore differences among populations. A more recent approach to analyse population differentiation is the hapFLK metric [179], which improves on single locus statistics by testing haplotype differentiation. hapFLK corrects frequency estimates, accounting for the genetic relationship between populations using Reynolds genetic distances.

Selection for a favourable allele of a gene increases the levels of linkage disequilibrium (LD) around the locus under selection, until recombination occurs to reduce the extent of LD [180]. Selection signatures can therefore be found by detecting regions of strong LD relative to their prevalence within a population [181,182]. Alleles at linked loci are referred to as haplotypes. Extended haplotype homozygosity (EHH) methods measure the decay of haplotype homozygosity as a function of genetic distance. The integrated Haplotype Score (iHS) [183] is calculated from the integrals of the observed decay of EHH for the ancestral and derived alleles surrounding the locus under selection. Divergence between values from the genomic average is indicative of selection. This approach requires phased data and knowledge of the ancestral state for each allele, and it has low power when one allele is at high frequency or fixed. Cross-population methods such as XP-EHH [182] and Rsb [184] calculate EHH profiles between two populations, removing the need to know the ancestral state. These methods have high power for detecting selective sweeps that have reached fixation. Selective sweeps generate runs of homozygosity (ROH) when both parents pass on the same haplotypes that are inherited from one generation to the next [185].

### 4.3. Local Ancestry Inference

Local ancestry inference (LAI) identifies the ancestors of each genomic region at the chromosome level. LAI is also described as local ancestry deconvolution or chromosome painting. Local ancestry information can help to understand fine scale admixture and the population genetic history, identify recent targets of selection, guide the selection of reference panels for genotype imputation, and improve the detection power of genetic association studies of admixed populations [184,186,187,188,189]. Identifying the ancestry of chromosomal segments in admixed individuals facilitates the accurate identification of the history of genetic variants under selection [188], particularly where adaptive introgression has fixed or nearly fixed regions of the genome with specific population ancestry [190].

Most approaches to profile local ancestry divide the genome into windows and assign ancestry to each window by comparing it against a reference panel [186,188,191,192,193,194,195]. New methods do not require the explicit definition of a reference population [196,197]. The most popular algorithms for LAI rely on hidden Markov models (HMM), an extension of a Markov chain, to identify the transformation of a genomic region from the reference, which is often not obvious [198]. These methods provide the posterior probabilities for each possible ancestry state at each ancestry-informative site along the chromosome [189,190]. The estimates obtained depend largely on reference populations; therefore, approaches to identify convergent signals of ancestry across multiple tests using different references have been developed [199].

LAI has been widely applied to identify adaptive introgression related to climatic stressors in livestock. Adaptive introgression from wild to domestic sheep of loci affecting climatic adaptation and resistance to pneumonia has been identified using LAI [143,199]. Using LAI and multiple-reference adjustments, ancestry components of indicine origin were found in cattle breeds from Central Italy that are associated with resilience to harsh environments and climatic conditions [200]. A region of indicine introgression into Italian local taurine breeds has been identified on BTA18 containing *KLHL36, USP10, KIAA0513* and *FAM92B*, all of which are related with residual feed intake [200]. This introgression could provide an adaptive advantage enabling animals to use low quality feed efficiently. 

Introgression of genes regulating the response to hypoxia from yak into Tibetan cattle that facilitated the adaptation of the latter to high altitude was also identified by LAI [201]. Similarly, adaptive introgression of genes related to oxygen transportation from Argali sheep to Tibetan domestic sheep may be a key factor conferring high-altitude resilience [202]. Local ancestry signals in African cattle have identified the genomic components of indicine cattle related to heat tolerance and water reabsorption, along with innate-immune resistance to tick and tick-borne diseases [203]. LAI tests have provided evidence of adaptive introgression between llama and alpaca for coat colour, fibre characteristics, and adaptation to high altitude and harsh environment [172].

### 4.4. Landscape Genomics

Landscape genomics explores the interaction between the genome and the environment to better understand evolution by combining landscape ecology and population genetics [204,205]. Two advances enabled landscape genomics to be realized. The first was the development of Geographic Information Systems (GIS) [206], which facilitated the overlay of diverse geo-referenced information, in this case genetic and environmental data. The second was the availability of large numbers of genetic markers, specifically single nucleotide polymorphisms, that are easily assayed. The development of the software MatSAM to compare a large number of allele frequencies with eco-climatic variables brought these two advances together as landscape genomics [207]. The MatSAM software [208] has been successfully used for landscape genomics analyses of plant and animal species, including sheep [207], goats [209] and fish [210]. These studies used GIS to store both genetic and environmental variables retrieved from open access databases to create gene–environment matrices that are processed by logistic regressions. Several software programs using different models have been developed for land-scape genomic analysis; improvements of these have an ever-increasing capability to efficiently analyse big data sets of genomic and environmental variables (see Box 5). 

Landscape genomics approaches were used to understand the genetic adaptation of South African goats, finding that climatic variables explained 17% of their overall diversity. Using SAM software (see Box 5 and [207]), 843 SNPs were identified that were associated with longitude, while LFMM software [211] found that 714 SNPs were associated with temperature and precipitation [212], with only one locus in common that included *DGKB*. These SNPs were close to genes involved in 205 biological pathways, all of which are potentially related to adaptation. Among the genes identified, several have been associated with thermoregulation in hot environments (e.g., *PLCB1*). In the analysis of a goat database of more than 1000 animals covering 33 Italian populations using landscape genomics methods and LFMM [213], identified many loci putatively associated with environmental variables, although there was no overlap in loci identified by each of the methods. Samβada identified 62 genes associated with temperature or precipitation; among these, *RYR3* has been associated with mean temperature and *ANK3* and *BTRC* with longitude [214]. The LFMM analysis identified four SNPs associated with Mean Diurnal Range and Mean Temperature. These SNP were near *NBEA*, located within a region involved with wool production in sheep [215], and *RHOBTB1*, which is known to be associated with meat quality in cattle [216]. As observed before, methods implemented in Samβada and LFMM produce non-overlapping results. The two software are suited to the analysis of population having specific genetic structure (see Box 5) and their use is suggesed as complementary rather than alternative tools. Colli et al. [217] applied landscape genomics software based on the SAM approach to analyse 43 European and West Asian goat breeds. Using AFLP markers, four loci were identified that were significantly associated with diurnal temperature range, frequency of precipitation, relative humidity and solar radiation. 

A landscape genomic analysis of 57 sheep breeds using the SAM approach found that the *DYMS1* microsatellite locus was associated with the number of wet days, which largely affects parasite load [207]. In an earlier study this locus was shown to be associated with parasite resistance [218].

Box 5 **Landscape Genomics Software.**

With the availability of increasing numbers of measures of environmental variables and an increasing number of genetic markers, the MatSAM software [208] was developed to process many simultaneous univariate association models. Samβada [213] is able to compute univariate and multivariate logistic regressions, integrate and make an intelligent selection of significant models, calculate pseudo R2, Moran’s I, and Geographically Weighted Regressions. This software has High Performance Computing (HPC) capacities to handle the large datasets created when several million SNPs, produced by high-throughput sequencing, are combined with hundreds of environmental variables. Samβada is also supported by R-SamBada [219], an R software package that provides a complete pipeline for landscape genomic analyses, from the retrieval of environmental variables at sampling locations to gene annotation using the Ensembl genome browser.

Other landscape genomics software include BAYENV [220], which uses the Bayesian method to compute correlations between allele frequencies and ecological variables, taking into account differences in sample size and population structure; LFMM [211,221], which identifies gene-environment associations and SNPs with allele frequencies that correlate with clines of environmental variables; and SGLMM [222], which extends the BAYENV approach [223] by using a spatially explicit model and calculating inferences with an Integrated Nested Laplace Approximation and Stochastic Partial Differential Equation (SPDE). BayPass [224] builds on BAYENV to capture linkage disequilibrium information. BAYESCENV [225] produces an F_ST_-based genome scan, taking into account environmental differences between populations. The latest version of LFMM [226] improves on both scalability and speed with respect to other GEA methods using a least-squares approach to estimate cofounders. Moreover, LFMM uses several categories of genomic data which are not restricted to genotypes.

Landscape genomics studies often use population genomics software (e.g., LOSITAN based on the FDist model [227,228]) to compare the sets of candidate loci obtained from different approaches: see BayeScan [229] and Bayenv [223]. A comparison of results allows for consolidation, as the accuracy of methods is known to differ (see, e.g., [213]). Samβada / R–SamBada [219] gives reliable results when the population structure is weak, while LFMM2 [226] is better suited to detect selection signatures in well-structured populations. Analyses of simulated data using, e.g., CDPOP [230] is usually advised to demonstrate the effectiveness of the method before moving to the analysis of empirical data (see, e.g., [211,213,231]). GEONOMICS, a Python package, performs forward-time, individual-based, continuous-space population genomic simulations on complex landscapes [232]. GEONOMICS includes several analytical steps using models of a landscape with one or more environmental layers (geotiff files as input), each of which can undergo environmental changes, as well as species having genomes with realistic architecture and associated phenotypes. Species undergo non-Wright–Fisher evolution in continuous space, with localized mating and mortality. The results produced are useful for a wide variety of theoretical and empirical purposes such as species conservation and management.

### 4.5. Artificial Intelligence and Machine Learning Approaches 

With advances in genomic technology and more sophisticated sensing systems, “big data” sets are being created and a large amount of data needs to be stored every day [233]. These data sets will potentially reveal changes in genomes that adapt animals to a wide range of conditions and environments. However, the information is a mixture of homogeneous and heterogeneous data types where the relationships among parameters may be hidden or difficult to identify. Artificial Intelligence (AI) and Machine Learning (ML) methods are increasingly used to extract information from this type of data to overcome the limits of traditional linear models (250, 251) (see Box 6). ML and AI have not yet been fully applied to study adaptation to climate change in livestock; however, the role of big data and machine learning will become increasingly important for modern farming [234].

ML methods have been used in the quest for regions associated with adaptation, in particularly to detect *de novo* mutations and selective sweeps for previously segregating variants in humans [235]. The S/HIC Deep Learning (DL) model has shown that most human mutations are neutral in populations, and that those conferring an adaptive advantage only rise in frequency when a change in the environment gives advantages to individuals carrying a particular mutation [236]. This approach has been used to identify genes associated with metabolism in a southern African ethnic groups using the SWIF(r) DL algorithm [237]. Variants of these genes arose thousands of years ago to store fat when food was scarce. 

There are a few examples of the use of ML in livestock genetics and breeding [196,238,239], and new DL genetic models are only just being tested [240,241,242,243]. The identification of SNPs directly associated with candidate genes affecting growth traits in Brahman cattle was more successful using ML Gradient Boosting Machine (GBM) than Random Forest statistical methods [241]. ML algorithms have been used together with RNA-Seq expression data to identify genes associated with feed efficiency in pigs, and to classify animals’ phenotypic extreme for residual feed intake [244].

Box 6 **Artificial Intelligence and Machine Learning.**

Artificial Intelligence (AI) uses algorithms that automate the decision process [245], while Machine Learning (ML) uses AI to automatically learn complex relationships and patterns in data [246,247]. ML algorithms may be unsupervised or supervised. The former explores the dataset structure without prior knowledge of data organization, while the latter uses prior knowledge to train the model and predict the outcome in a test dataset [248]. ML algorithms are adapted to explore nonlinear relationships [249]. Deep learning (DL) creates multiple processing layers (neural networks), which mimic the structure of a human brain, to extract information and learn from the input data. DL is being used to discover intricate structures in large datasets [246,250]. However, the neural network models are a “black box” as they are hidden as they develop. Tools are being developed to dissect the layers of the models developed to understand the neural network process; one example are the saliency maps [251,252].

ML methods mainly focus on prediction, while classical statistical methods rely on inference [253]. ML has been used to recognize the location of specific sequence elements (i.e., splice sites, promoters, etc.) and to combine genomic elements to identify and annotate genomic features, e.g., to identify UTR, introns, and exons, and to functionally annotate genes [235]. For example, S/HIC (https://github.com/kern-lab/shIC) is an ML classifier developed to detect targets of adaptive natural selection from whole genome sequencing data.

Efficient DL software tools such as Tensorflow and Keras Python libraries, and the availability of supercomputing using graphics processing unit technology (GPU), have opened the way to the integration of multi-omics big data with environmental variables.

**Table 1:** Software for genome-wide analyses.

Software	Method	Application	Ref.	Link
Arlequin	Tajima’s D	Selection signatures	[254]	http://cmpg.unibe.ch/software/arlequin35/
BayeScan	F_ST_	Selection Signatures, Landscape genomics	[229]	http://cmpg.unibe.ch/software/BayeScan/
Bcftools	ROH	Selection signatures	[255]	https://github.com/samtools/bcftools
DnaSP	Tajima’s D and Fay and Wu’s statistic	Selection signatures		http://www.ub.edu/dnasp/
Hapbin	EHH	Selection signatures	[256]	https://github.com/evotools/hapbin
hapFLK	hapFLK	Selection signatures	[179]	https://forge-dga.jouy.inra.fr/projects/hapflk
HierFstat (R package)	F_ST_	Selection signatures	[257]	https://cran.r-project.org/web/packages/hierfstat/index.html
KING	ROH	Selection signatures	[258]	https://www.kingrelatedness.com/
PLINK	F_ST_, ROH	GWAS, Selection Signatures	[259]	https://www.cog-genomics.org/plink/2.0/https://www.cog-genomics.org/plink/
PopGenome	Tajima’s D	Selection signatures	[260]	https://cran.r-project.org/web/packages/PopGenome/index.html
PoPoolation	Tajima’s D	Selection signatures	[261]	https://sourceforge.net/p/popoolation/wiki/Main/
rehh (R package)	EHH	Selection signatures	[262]	https://cran.r-project.org/web/packages/rehh/index.html
Selscan	EHH	Selection signatures	[263]	https://github.com/szpiech/selscan
VariScan	Tajima’s D	Selection signatures	[264]	http://www.ub.edu/softevol/variscan/
VCFtools	F_ST_, Tajima’s D	Selection signatures	[265]	http://vcftools.sourceforge.net/
EMMAX	GWAS based on variance component model	GWAS	[266]	http://genetics.cs.ucla.edu/emmax
GCTA	GWAS based on genome-wide complex trait analysis	GWAS	[267]	http://gump.qimr.edu.au/gcta
BayesR	Bayesian mixture model	GWAS	[268]	http://www.cnsgenomics.com/software/
MatSAM	Logistic regression	Landscape genomics	[208]	www.econogene.eu/software/sam/
Samβada, R.SamBada (R package)	GEA based on logistic regression/spatial autocorrelation	Landscape genomics	[213,219]	https://github.com/Sylvie/sambada/releases/tag/v0.8.3https://cran.r-project.org/package=R.SamBada
BAYENV	GEA based on Bayesian regression	Landscape genomics	[220]	https://gcbias.org/bayenv/
LFMM2 (R package)	GEA based on latent factor mixed models	Landscape genomics	[221,226]	https://bcm-uga.github.io/lfmm/
SGLMM	GEA based on allele-environment association analysis	Landscape genomics	[222]	-
BayPass	GEA corrected for the covariance structure among the population allele frequencies	Landscape genomics	[224]	http://www1.montpellier.inra.fr/CBGP/software/baypass/
BAYESCENV	GEA based on F_ST_ genome-scan	Landscape genomics	[225]	https://github.com/devillemereuil/bayescenv
LOSITAN	F_ST_	Landscape genomics	[227]	https://mybiosoftware.com/lositan-1-0-0-selection-detection-workbench.html
PCAdmix	Supervised LAI	Local Ancestry Inference	[186]	https://sites.google.com/site/pcadmix/home
Tractor	LA-aware regression model	Local Ancestry Inference	[187]	https://github.com/eatkinson/Tractor
LAMP	LAI accounting for recombination	Local Ancestry Inference	[188]	http://lamp.icsi.berkeley.edu/lamp/
MOSAIC (R package)	Unsupervised LAI	Local Ancestry Inference	[193]	https://maths.ucd.ie/~mst/MOSAIC/
RFMix	LAI based on conditional random field	Local Ancestry Inference	[194]	https://github.com/slowkoni/rfmix
Loter	LAI for species other than humans	Local Ancestry Inference	[195]	https://github.com/bcm-uga/Loter
GHap (R package)	Unsupervised LAI	Local Ancestry Inference	[196]	https://cran.r-project.org/package=GHap
PSIKO2	Unsupervised LAI	Local Ancestry Inference	[197]	https://www.uea.ac.uk/computing/psiko
SWIF(r)	Probabilistic method to detect selective sweeps	Deep Learning	[237]	https://github.com/ramachandran-lab/SWIFr

## 5. Conclusions

To maintain animal welfare and as a consequence productivity and production efficiency, breeds have to be well adapted to the environmental conditions in which they are kept. Rapid climate change inevitably calls for the use of various countermeasures to manage animals appropriately. Temperature mitigation methods (shaded area, water wetting, ventilation, air conditioning) are possible solutions; however, these can only be used when animals are kept in shelters and are not applicable to range-type farming systems. Most structural solutions to control the environment of animals have a high cost, and many have energy requirements that further contribute to climate change. Therefore, addressing livestock adaptation by breeding animals that are intrinsically more tolerant to extreme conditions is a more sustainable solution. Decreasing stress and increasing animal welfare is important for farmers and the general public. Animals stressed by high temperatures may be less able to cope with other stressors such as pollutants, dust, restraint, social mixing, transport, etc., that further affect welfare and productivity. Innovation in sensors and linking these into the “internet of things” (IoT) to collect and exchange data is increasing our ability to record environmental variables and animal welfare status and provide input to systems dedicated to the control of environmental conditions and provision of early warning of discomfort in individual animals. In the longer term, collecting such data will contribute to understanding the genetics underpinning tolerance and adaptation to environmental and other stressors in order to select animals better suited to different conditions. The resulting increase in efficiency will have additional benefits in terms of reducing greenhouse gas emissions, particularly methane from ruminants, which currently make a significant contribution to climate change.

Breed substitution by introducing breeds known to have particular resilience, e.g., to drought, temperature extremes or disease, may be a solution. This approach would facilitate a rapid response to climate change, although it is not ideal as breeds more tolerant of hot climates generally have low productivity. Additionally, imported breeds may not adapt to local conditions such as available feed resources and disease challenge. 

Crossbreeding between highly productive and heat tolerant breeds is an approach that is currently used in tropical areas including Australia, the southern USA and Brazil, where crossing productive taurine breeds with heat adapted indicine breeds facilitates improved production in extreme conditions. Selection of these cross-bred populations has produced stable breeds that show good productivity and adaptation, such as the Brangus from the USA [269] and the Australian Droughtmaster [270]. O’Neil et al. [271] have reviewed the use of crossbred lines in tropical high tick challenge areas of Australia. However, crossbreeding programs should be properly planned, organised and monitored, as indiscriminate crosses may cause the genetic erosion of local breeds and the loss of their adaptation.

Accelerated selection for thermal tolerance and resilience to new endemic diseases is also a possible sustainable solution. In this case, genomics plays a key role together with phenotype recording and the collection of epidemiological and environmental data. Research is approaching the challenging task of identifying genes having adaptive value using a range of methods, including those described in this review. Specific variants of major genes exist in local genetic resources, as demonstrated by the SLICK mutation associated with heat tolerance. However, identifying causal genes and variants is difficult, requiring large data sets which are often not available or affordable for livestock, and a focused effort to refine and test candidate genes. Therefore, most studies have simply localized genetic effects to chromosomal regions or quantitative trait loci (QTL) in genome-wide association studies. Additionally, it is now clear that most adaptation traits have complex genetic control, making the genetic basis difficult to unravel. Nevertheless, markers having significant effects can be used in selection programmes using marker assisted selection or by weighting particular SNPs within QTL regions in genomic selection estimates. Although genomics is presently only scratching the surface of the control mechanism of these traits, comparison between methods, studies, breeds and even species is starting to reveal that morphology, energy and lipid metabolism, and the immune system are key factors in adaptation, with some genes being consistently identified as carrying variants modulating adaptation. The identification of these genes confirms the importance of the conservation of local genetic resources as reservoirs of useful alleles. The evaluation and improvement of these breeds or the transfer of adaptive variants into highly selected breeds are the next steps to better match livestock to harsh conditions while maintaining productivity. These steps may be accelerated by marker-assisted or genomic selection, and even more rapidly by novel tools such as gene editing where such approaches are socially accepted. Parallel breeding for adaptation to climate change and the mitigation of the impact of livestock on climate change is probably the hardest challenge that the livestock sector has ever faced, but it is now urgent. The challenge can only be won if research, industry, decision makers and funders join forces with the objective of satisfying the rights future generations to a healthy diet and a clean planet. 

## Data Availability

Not applicable.
